# Small Bowel Gastrointestinal Bleeding Diagnosis and Management—A Narrative Review

**DOI:** 10.3389/fsurg.2019.00025

**Published:** 2019-05-16

**Authors:** B. Murphy, D. C. Winter, D. O. Kavanagh

**Affiliations:** ^1^Department of Colorectal Surgery, University Hospital Tallaght, Dublin, Ireland; ^2^Department of Colorectal Surgery, St. Vincent's University Hospital, Dublin, Ireland

**Keywords:** small bowel bleeding, gastrointestinal hemorrhage, GI hemorrhage, obscure gastrointestinal (GI) bleeding, occult gastrointestinal bleed

## Abstract

**Background:** Small bowel bleeding accounts for 5–10% of all gastrointestinal bleeding. Despite advances in imaging, endoscopy and minimally invasive therapeutic techniques, its diagnosis and treatment remains a challenge and a standardized algorithm for approaching suspected small bowel bleeding remains elusive. Furthermore, the choice of investigation is subject to timing of presentation and accessibility to investigations. The aim of this study was to construct a narrative review of recent literature surrounding the diagnosis and management of small bowel bleeding.

**Methods:** A literature review was conducted examining the database pubmed with the following key words and Boolean operators: occult GI bleed OR mesenteric bleed OR gastrointestinal hemorrhage OR GI hemorrhage AND management. Articles were selected and reviewed based on relevance to the research topic. Where necessary, the full text was sought to further assess relevance.

**Results:** In overt GI bleeding, CT angiography and red cell scintigraphy are both feasible and reliable diagnostic imaging modalities if standard endoscopy is negative. Red cell scintigraphy may be advantageous through detection of lower bleeding rates but it is subject to availability. Overt bleeding and a positive CT angiogram or red cell scan improves the diagnostic yield of formal angiography ± embolization. Video capsule endoscopy or double balloon endoscopy can be considered in occult GI bleeding following normal upper and lower endoscopy.

**Conclusions:** Small bowel bleeding remains a rare but significant diagnostic and therapeutic challenge. Technological advances in diagnostics have aided evaluation but have not broadened the range of therapeutic interventions.

## Introduction

Small bowel bleeding accounts for 5–10% of all Gastrointestinal (GI) bleeds (1). GI bleeding can be assumed to be of small bowel origin once meticulous upper and lower GI endoscopy has been completed without an identifiable source. Anatomically, it may be defined as bleeding distal to ampulla of Vater and proximal to the ileocaecal valve (1).

Terms frequently in use in relation to GI hemorrhage include acute or **overt** GI bleeding when a patient has objective malaena, hematochezia or hematemesis and occult GI bleeding when a patient may present with more insidious symptoms including iron deficiency anemia with or without a positive fecal occult blood stool test. These should not to be confused with the term obscure GI bleeding which is now largely historical and associated with an era predating modern diagnostic imaging and endoscopic techniques.

Due to the difficulty in direct visualization of the small bowel prior to recent endoscopic advancements, there has been much discussion regarding the most appropriate management of small bowel bleeding. This has perpetuated a lack of clarity regarding both appropriate use and sequence of investigations among treating health care professionals. In this review, we will address recent literature surrounding this topic, mainly focusing on diagnosis of small bowel GI bleeding and therapeutic options.

## Aetiology

Causes of small bowel bleeding may be categorized as vascular, inflammatory, iatrogenic, diverticular or secondary to tumors (2). Some of the most common causes are summarized in Table 1.

**Table 1:** Common causes of small bowel bleeding.

**Vascular**	**Inflammatory**	**Iatrogenic**	**Diverticular**	**Tumor**
Angiodysplasia	Crohn's Disease	Medications i.e., Non-steroidal anti-inflammatory drugs (NSAIDS)	Meckel	Gastrointestinal stromal tumor (GIST)
Dieulafoy lesions	Intestinal tuberculosis	Radiation	Small bowel diverticula	Lymphoma
Arteriovenous malformation (AVM)	Behcet's disease	Surgical/Procedure related	–	Adenocarcinoma Polyps Lipoma

Causative factors may be further stratified based on age and it is essential to consider this when formulating a management plan. Angiodyplasia, malignancy and ulcers are more likely to occur in older patients whereas in younger patients, small bowel bleeding is more likely to be caused by inflammatory bowel disease (IBD), Dieulafoy lesions or a Meckel's diverticulum (2). Angiodysplastic lesions are thought to be the most commonly detected lesions in the small bowel and are found in ~40% of patients with bleeding (3). Other vascular lesions, such as Dieulafoy's lesions and varices may be detected in up to 20% of patients (4) and similarly, ulcers/erosions can be anticipated in up to 30% (5). Tumors including small bowel malignancy and polyps are found in ≤5% (6). Details pertaining to the patient history are important to note when trying to determine the etiology of a small bowel bleed. A history of any clotting abnormality and medications including antiplatelets, anticoagulants and non-steroidal anti-inflammatory drugs (NSAIDs) is essential to elicit. Knowledge of co-morbidities, such as valvular heart disease which may predispose to Heyde's syndrome is also paramount where relevant as angiodysplasic lesions are a feature of this condition (2).

## Diagnosis

### Repeat Endoscopy

Fifteen to Twenty percent of patients with suspected small bowel GI bleeding however will have an upper or lower GI source that has been missed on initial endoscopy (7). Lesions may be missed at first endoscopy for a variety of reasons including but not limited to poor visibility due to active bleeding/food debris and poor or no bowel preparation in the case of lower GI endoscopy. The diagnostic yield on repeat OGD for GI bleeding has been estimated up to 29% and colonoscopy at up to 6% by the American Society of Gastrointestinal Endoscopy (ASGE) (8). The American Gastrointestinal Association (AGA) thus advocates repeating GI endoscopy in patients in whom a cause has not been found at first look.

It is at the discretion of the clinician and case dependent, but a repeat OGD alone can be considered first in lieu of double endoscopy due to a higher diagnostic yield and the lack of bowel preparation which may impose further physiological stress on an unwell and often elderly patient (9).

Some institutions advocate initial push enteroscopy i.e., an extended OGD using a longer endoscope or pediatric colonoscope instead of Upper GI endoscopy as this may be more cost-effective (10). This practice is unfeasible however in hospitals which do not habitually provide this service. Furthermore, it has been found that most lesions identified at push enteroscopy as a “second look procedure” would have been visible on a repeat standard OGD (11).

### Computed Tomography (CT)

Cross sectional imaging for small bowel bleeding includes conventional CT abdomen and pelvis, mesenteric CT angiography (CTA), CT enterography (CTE) and Magnetic resonance enterography (MRE). Due to the limited availability and time constraints of MRE, CT has been generally favored as an acute-phase diagnostic tool.

CT imaging is often used when endoscopic examinations have failed to identify a source of bleed or are unfeasible to perform. Its role is primarily diagnostic i.e., to determine the presence, location and ideally etiology of the bleed. A conventional CT abdomen/pelvis is unlikely to demonstrate subtle findings but may show a tumor or mass which could be causative in a GI bleed or acute inflammation (12).

To examine the small bowel for **occult** bleeding, CT enterographic techniques may be considered if available at the treating clinician's institution. The sensitivity of CTE in identifying the causes of small bowel GI bleeding varies among institutions. In a meta-analysis of 18 studies, CTE had a pooled diagnostic yield of 40% compared to other modalities, such as Capsule Endoscopy (53%) (13). Some single centers, however, have reported figures as high as 88% (14).

CTE can be more accurate in detecting small bowel tumors and Crohn's disease than detecting Meckel's diverticula and angiodysplasia which may explain to some extent the variability in success rates (14). Angiodysplasia is characterized by dilated submucosal veins occasionally accompanied by a dilated feeding artery and draining veins in larger lesions. On CTE, it may appear as a nodular enhancing lesion within the bowel wall. The presence of the aforementioned vascular findings may implicate the diagnosis, but these are more commonly seen in colonic angiodyplasia and less frequently in the small bowel. An uninflamed Meckel's diverticulum is difficult to detect at CT because as a true diverticulum containing all bowel wall layers, it appears similar to normal small bowel unless a characteristic blind end is noted (15, 16).

The diagnosis of active or overt GI bleeding can be made on CT Angiogram following extravasation of contrast material into the bowel lumen. There is no administration of oral contrast which is particularly appropriate in unstable patients, and additionally, oral contrast can dilute extravasation of intravenous (IV) contrast in the bowel lumen making a potential bleeding point subtler in appearance. Both arterial-phase and venous-phase scans can be utilized as slower bleeding may be visible only on the latter. Hyperattenuating intraluminal material on a pre-enhanced scan may indicate acute hematoma and, therefore, a site of recent bleeding (17).

Bleeding rates of 0.5–1.0 mL/min have traditionally been considered necessary to demonstrate bleeding on CTA; this is equivalent to 2–3 units of blood loss over 24 h. A meta-analysis of nine studies with 198 patients with overt GI bleeding showed CTA had a pooled sensitivity of 89% and specificity of 85% in diagnosing acute bleeding throughout the GI tract (18). Likewise, a negative CTA may be useful for guiding further management as the likelihood of a subsequent positive angiography following this is very low. If bleeding is persistent, further work-up may be necessary but conservative management of these patients will often be successfully providing the patient remains hemodynamically stable and sinister pathology is not suspected (19).

### Nuclear Medicine

The most frequently used Nuclear Medicine (NM) scan for the detection of acute GI bleeding is 99 m Technetium labeling of autologous blood. Typically, 4 mls of the patient's blood is removed, labeled and then reinjected into the patient. The patient's abdomen is thereafter scanned for 1 h during which time a GI bleed is detected as intraluminal or extraluminal activity moving in a linear or curvilinear fashion.

Red cell scans can detect bleeding rates as low as 0.1 ml/min demonstrating an advantage to CT angiography. A disadvantage is that it may only provide an approximate location of the bleeding point unlike CT which is more precise. Along with CT imaging, it lacks a therapeutic component (20–22).

Further limitations include lack of immediate availability, notably during on call hours, and subjectivity to false positive localization of a bleed due to tagged blood moving within the GI lumen during peristalsis (23) or position changes in the context of various non-GI pathologies e.g., uterine fibroids (24). The overall positive yield of red cell studies is reported to be ~50% (23).

A prospective study from Zink et al. (25) examining a comparison of Red Cell Scintigraphy and CTA for lower GI bleeding demonstrated a positive i.e., localization of bleeding point in 46% of patients and 27% of patients, respectively. Similar prospective data are lacking for small bowel bleeding.

A further advantage of red cell scintigraphy is its usefulness in directing the timing, appropriateness and likely success of subsequent angiography. While precise localization of the bleeding point is often suboptimal compared to CT, arterial angiography is more likely to be successful following a positive bleeding scan; a positive red cell scan has been shown to increase the likelihood of a positive angiogram (26, 27).

A study by Ng et al. (28) showed that performing a formal angiogram immediately after a positive early phase red cell scan (i.e., positive for bleeding within 2 min of infusion labeled red cells) resulted in localization of bleeding in 67% of cases. In contrast, performing angiography immediately after a positive late phase bleeding scan (scan positive for bleeding after 2 min of infusion of labeled red cells) had a lower yield of 7%.

Considering these factors, some expert bodies including the American Society for Colorectal Surgery (ASCRS) advocate red cell scintigraphy as the first-line diagnostic scan of choice (29). It appears to be generally accepted however that the perceived advantage of red cell scintigraphy in detecting a bleeding point should be counterbalanced with the speed, availability and more accurate localization of CTA. CTA is therefore widely acknowledged as a reliable and acceptable choice in the absence of red cell scan availability or feasibility.

### Angiography

Formal angiography has the advantage of being potentially both diagnostic and therapeutic when performed. The yield of angiography is largely dependent on the rate of bleeding as suggested by the higher positivity after an immediately positive red cell scan (28).

Overall, the diagnostic yield has been reported between 27 and 77%, but a higher yield of 61–72% has been observed in active or overt bleeding in comparison to <20% in those with inactive or suspected occult bleeding. In keeping with this, further predictors of positive angiography include hemodynamic instability and the requirement of ≥5 units RCC transfusion (9).

Success rates may vary. In one study by Koh et al. (30), 48 invasive angiograms were performed following positive CTA. Of these, 25 (52.1%) were positive and underwent embolization with Gelfoam or coiling with a clinical success rate of 68.4% and no significant complications. Interestingly, there were no significant differences noted between the groups with positive and negative angiography in terms of age, gender, co-morbidity, hemoglobin, and coagulation profile.

Complications may occur in up to 10% of patients undergoing angiography, most notably renal impairment, bowel infarction, infections and bleeding at the catheter site (31). These interventions however have seen significant advancements in recent years with the advent of su*per-se*lective angio-embolization resulting in higher success rates and fewer complications. Some studies report up to 99% technical success rate and 71–79% clinical success rate (32, 33).

### Provocative Angiography

The concept of provocative angiography i.e., provocation of bleeding with use of anticoagulants, vasodilators and thrombolytic agents, has been proposed in cases where a bleeding point cannot be identified by more conventional means (34). The yield has been reported as 29–67% and has been shown in a few small studies to be increased compared with standard angiography (35, 36).

Expectedly, provocative angiography is associated with an increase in bleeding complications with some studies reporting catheter site bleeding in up to 17% (37). While few studies appear to report catastrophic bleeding events following provocative angiography, it is advisable to consider that these figures represent a small number of studies performed on highly selective cases in experienced centers. Early involvement of the surgical team is prudent if this is undertaken in the event of precipitated major hemorrhage.

### Video Capsule Endoscopy (VCE)

Prior to the introduction of VCE, options for complete small bowel imaging were confined to imaging studies with limited diagnostic capability and intra-operative Endoscopy (IOE), which can have significant morbidity and cost implications (38).

The capsule is 11 × 26 mm. It takes two *images per second generating* 14,400–72,000 images. VCE is contraindicated if there is suspected bowel obstruction as this may lead to capsule retention. This is an infrequently encountered issue in patients with occult GI bleeding; however, if there is suspicion of underlying mass, a dissolvable patency capsule may be used initially to assess the risk of retention (39, 40).

In one single center study by Goenka et al. (41), from 385 patients investigated for small bowel bleeding, 284 (74%) had some lesion detected by VCE. In 222 patients (58%), definite lesions were detected that could irrefutably explain the presentation. Patients with overt GI bleeding for <48 h before VCE had the highest diagnostic yield (87%). This was significantly greater than that in patients with overt bleeding over 48 h before VCE was undertaken (68%). A positive result in occult GI bleeding was lower again at 59%. These findings suggest that an **overt** GI bleed with shorter intervals between the bleeding episode and performance of VCE increases the diagnostic yield.

Compared with alternative procedures for occult small bowel bleeding, capsule endoscopy appears to be a safe and effective choice, with an overall complication rate of 1–3% (42). A 2011 meta-analysis of 10 studies comparing VCE with double balloon enteroscopy demonstrated a diagnostic yield of 62 vs. 56%, respectively (43).

While the sensitivity of capsule endoscopy may exceed that of other diagnostic modalities, the specificity may be inferior to that of CT and push enteroscopy, ranging from 49 to 84% and thus potentiating false positive readings (44, 45).

Furthermore, mucosal views may be limited during capsule endoscopy due to a variety of factors including intraluminal material and rate of capsule progression (46). Studies assessing mucosal visualization in VCE using the ampulla of Vater as a landmark have reported low rates of identification ranging from 8 to 60% (47, 48).

Capsule endoscopy is also time-consuming and costly. A single procedure may produce between 14,400 and 72,000 frames of which only one may demonstrate a bleeding point (49). New software has being developed as a result to shorten the reading time for capsule endoscopy. This varies from detection of red pixels to selecting out one single representative frame for every ten. The sensitivity varies but some reports have suggested comparable results to standard reading (50).

Capsule endoscopy may incur more expense than standard endoscopy. A study from Ontario found that in cases of occult GI bleeding, the cost implications of performing capsule endoscopy as an adjunct to push enteroscopy in all cases of negative upper and lower endoscopy would increase annual costs by ~$1.5 million Canadian dollars (51). Further studies advocate however that this cost is justified when one considers the cost implications of repeat standard endoscopic procedures and recurrent admissions in the absence of capsule investigation on index presentation (52).

### Double Balloon Enteroscopy

Double balloon enteroscopy was developed in 2001 in Japan. Latex balloons are attached to the tip of the endoscope and overtube and inflated and deflated with air from a pressure-controlled pump system (53, 54).

The endoscope is advanced using the balloon and overtube in a “push and pull” maneuver to pleat the small bowel over the scope and visualize the mucosa. This can either be performed via the mouth or anus. The entire small bowel may be inspected with this procedure with studies from Japan reporting 86% entire small bowel visualization rate (55). In Western countries, this figure is generally reported as much lower (5%) (56). The reason for this is marked discrepancy is largely unclear but may be accounted for by center experience and quality of reporting. Unlike other endoscopic procedures, there are no current guidelines for threshold numbers to be performed to achieve competency in DBE. Like every procedure, there is likely an attached learning curve which may partially account for these contrasting figures. While an exact number of cases needed to achieve competency has not yet been validated, studies have reported decreased procedure times after 10 cases (57) and increased diagnostic yield from 58 to 86% comparing the first and last 50 cases in a 200-case series (58).

The most significant advantage of double balloon endoscopy over the video capsule endoscopy is that therapeutic interventions may be performed at the time of procedure. Therapeutic interventions can be performed for up to 27% of patients undergoing DBE for occult GI bleeding for pathologies not amenable to conventional endoscopic therapy, avoiding a potential bowel resection (56). It can facilitate surgical resection by allowing tattooing or endoscopic clipping of an otherwise impalpable mucosal or subcentimeter lesion.

Significant complications include perforation and bleeding which can arise in 1% of diagnostic procedures (59) and acute pancreatitis which may occur in 2–8% of DBE (60). The cause of pancreatitis is unclear but it is hypothesized that it may be due to prolonged mechanical stress due to repeated stretching by the endoscope or an increase in intraluminal duodenal pressure, leading to reflux of duodenal content into the pancreatic duct (6, 61).

## Treatment

### Medical Management

Either intravenous or oral Iron supplementation may be used in occult GI bleeding to maintain hemoglobin levels (48, 62). In more acute or overt bleeding, red cell transfusion should be used where necessary. Early consultation with a hematology service is advised, particularly in patients on anticoagulants or in whom massive blood loss is anticipated or suspected.

GI bleeding will occur in 1–3% of patients taking anticoagulant therapy (63). Risk factors for patients on anticoagulation for bleeding include age, comorbidity, use of NSAIDs or antiplatelets and previous GI bleeding (64). While cessation of anticoagulant or antiplatelet therapy either temporarily or indefinitely is routinely performed and seems like logical decision-making, holding these agents does not appear to lower the risk of recurrent bleeding, interestingly (65, 66).

Hormonal therapy with exogenous estrogens and progestogens has been utilized previously to treat small bowel GI bleeding but have generally been shown to have no role in its treatment (67). Somatostatin analogs, such as octreotide and lanreotide have also been used for small bowel bleeding as they inhibit angiogenesis, decrease splanchnic flow and improved platelet aggregation. Studies have demonstrated a reduction in transfusion rates following use of these agents (68, 69). Similarly, a meta-analysis from 2014 shows benefit from the use of somatostatin analogs in patients with angiodysplasia but not hormonal therapy (70).

Despite a harrowing past in obstetric medicine, Thalidomide has been used successfully to treat patients with refractory GI bleeding. It is an angiogenesis inhibitor and is therefore most effective in treating patients with vascular etiology, such as angiodysplasia and AVMs. In a randomized controlled trial by Ge et al. (71), 55 patients with recurrent GI bleeding secondary to angiodysplasia either received thalidomide 100 mg once daily or iron 400 mg once daily for 4 months. The primary endpoint was a reduction in bleeding episodes by ≥50% in 1 year. The response rate was 71.4% in the thalidomide group compared with 3.7% in the iron group.

Tranexamic acid has been shown to reduce mortality in bleeding trauma patients (72). A systematic review has found that it can reduce the probability of receiving a blood transfusion in up to 33% of acute GI bleed patients (73). The HALT-IT study is an ongoing multi-center randomized controlled trial assessing patient outcomes following early tranexamic acid administration compared to placebo in acute GI bleeding (74). Certainly, due to its widespread adoption and evidence basis in the trauma setting, the results of this trial are highly anticipated.

### Interventional Radiology and Endoscopy

The principals of IR management with embolization have been discussed previously in the diagnostic section. To recapitulate, the likelihood of having a positive angiogram is increased in cases of overt bleeding, a positive CTA or early phase Red Cell Scan. If a bleeding lesion is noted on angiography, success rates have been reported in 60–90% of cases. The advent of newer techniques and superselective embolization has further improved success rates and decreased the risk of bowel infarction (75, 76).

Endoscopic therapy depends on the etiology of the bleeding. As the most frequently encountered pathology, angiodysplastic lesions are treated typically with Argon Plasma Coagulation to good initial effect but rebleeding rates are high. A meta-analysis of 623 patients with a mean follow-up of 22 ± 13 months demonstrated recurrence i.e., rebleeding in 45% of treated small bowel angiodysplastic lesions (70).

### Surgery

Diagnostic laparoscopy or laparotomy may be considered in small bowel GI bleeding where no other modality has demonstrated a source. Furthermore, it may be utilized when a bleeding point has been identified but is not amenable to endoscopic/radiological therapy or mandates resection e.g., Meckel's diverticulum or mass lesion.

Intraoperative endoscopy (IOE) may be undertaken simultaneously via a small bowel enterotomy. Localization of a bleeding point at IOE can be made in 58–99% of cases according to published data with a therapeutic yield of 48–94% (77). In a study by Hartmann et al. (78), 47 patients with suspected small bowel bleeding and negative capsule endoscopy studies underwent IOE. The diagnostic yield was 73% overall; ranging from 100% in patient with ongoing overt bleeding, 70% in previous overt bleeding, and 50% in occult bleeding.

Occasionally, a combination of IR and surgery may be successful in the management of GI bleeding. Concerning angiodysplasia and AVMs of the GI tract, recurrent hemorrhage is common and bleeding may persist following initial hemostatic measures. In the case of recurrent bleeding, a small bowel vascular lesion may be palpable at surgery if an embolization coil has been placed pre-operatively, aiding accurate localization and resection (79).

### Future Considerations

It is discouraging that despite the evolution of radiological and endoscopic investigations in recent years, localization of a bleeding point remains such a challenge. Newer technology, such as biosensor placement may prove promising in this regard. Thompson et al. (80) have experimented in Harvard regarding sensor placement in a porcine model. This detects acute bleeding and notifies the treating physician immediately of same. This may aid diagnosis considering that imaging and endoscopic modalities have improved positive yield when performed closer to the time of active bleed. Applying this to clinical practice, future interventions may include endoscopic probe placement in the distal duodenum and terminal ileum to narrow down the site of bleeding and thus guide further management.

## Conclusion

With the advent of novel endoscopic and radiological techniques, the diagnosis and treatment of small bowel bleeding has evolved, however it nevertheless continues to pose a challenge for the treating clinician. The unabating lack of one unifying diagnostic modality with a desirably high sensitivity and specificity is integral to this challenge.

When approaching cases of small bowel hemorrhage, consideration should be made for differentiating between overt and occult bleeding as this will influence diagnostic yield and help guide appropriate management. Hemodynamic status must also be factored into decision making as a transfusion-dependent unstable patient is significantly more likely to have a positive angiogram ± embolization than a stable patient with chronic occult bleeding. A thorough patient history can also aid ascertainment of the etiology in addition to basic demographics including age.

It is imperative to remain continually mindful that small bowel bleeds are relatively infrequent and will only represent an estimated 5% of GI bleeding overall. Consideration should therefore always be given to repeat upper ± lower GI endoscopy if an identifiable source is not found at first look.

Medications, such as somatostatin analogs and thalidomide may be considered in the absence of contraindication to decrease the risk of rebleeding as adjuncts to interventional therapies.

**Figure 1:**
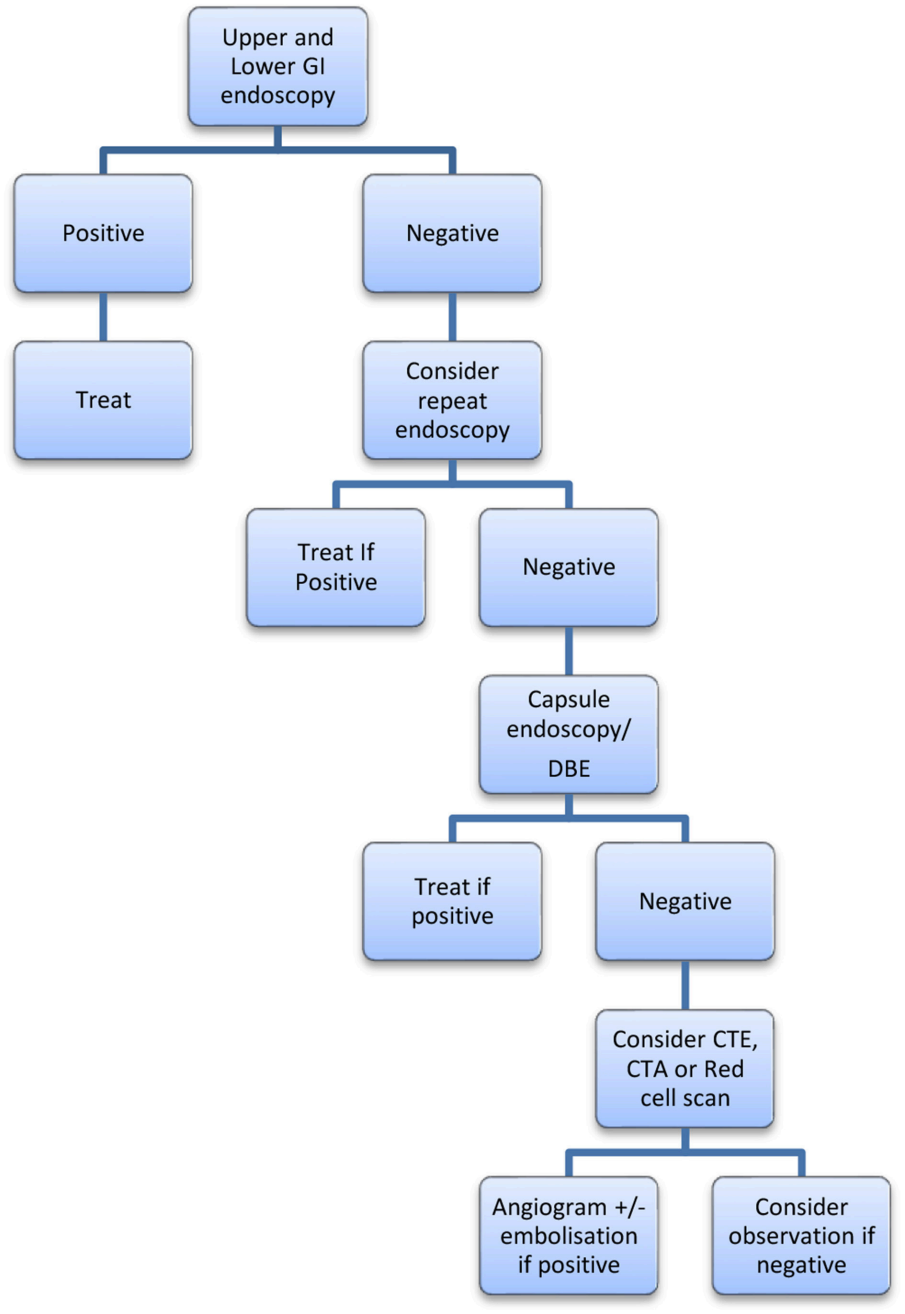
Proposed algorithm for occult GI bleeding.

**Figure 2:**
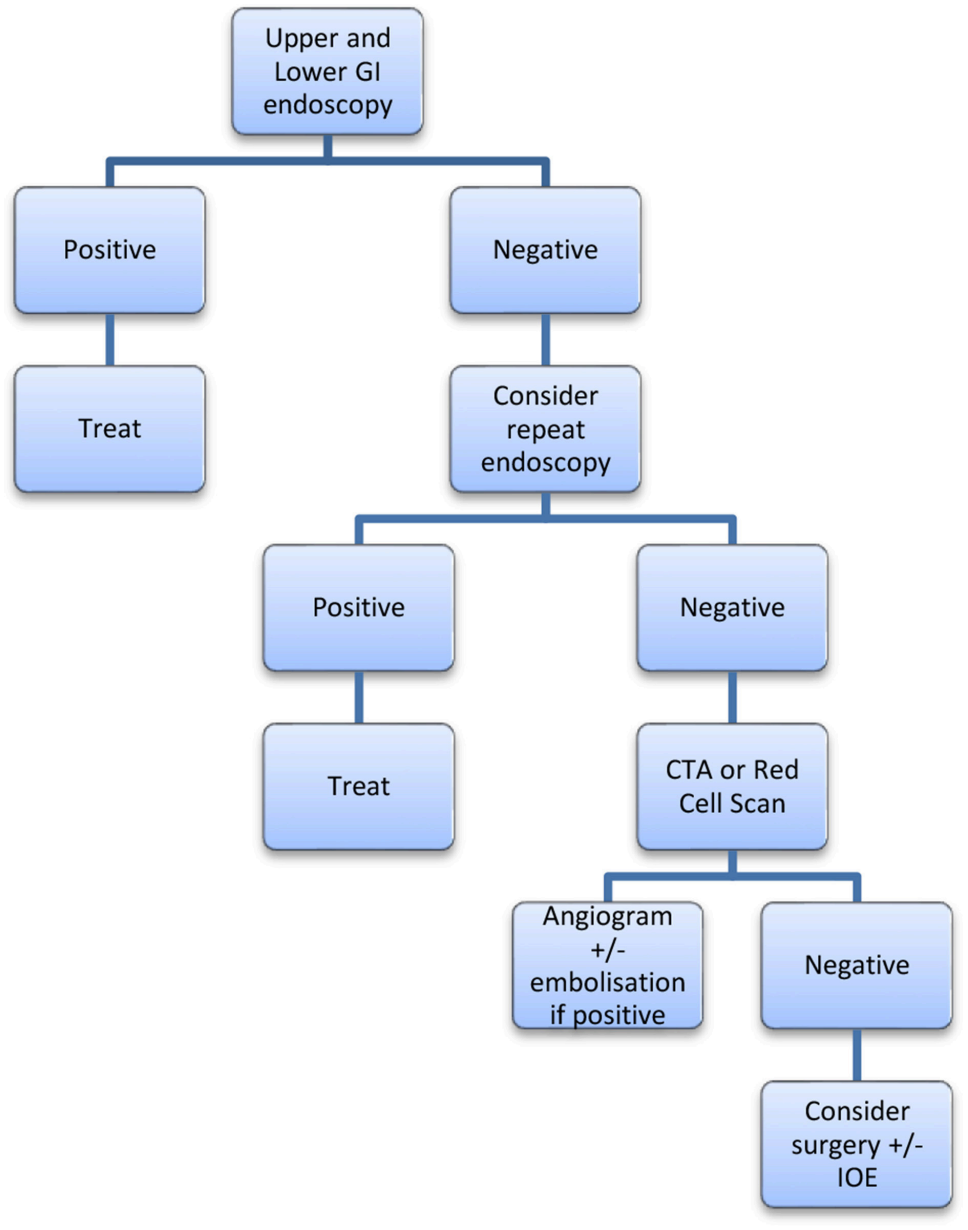
Proposed algorithm for Overt GI bleeding.

We propose treatment algorithms for overt and occult suspected small bowel bleeding (Figures 1, 2). These are guides to a treatment approach supported by the best available evidence albeit often level III. We acknowledge that this is subject to both availability and accessibility of the relevant diagnostic interventions at the treating clinician's institution.

## Author Contributions

BM authored main text. DW provided editorial advice. DK co-authored main text and provided editorial advice.

### Conflict of Interest Statement

The authors declare that the research was conducted in the absence of any commercial or financial relationships that could be construed as a potential conflict of interest.
